# Officers of N. Y. State Medical Society

**Published:** 1848-03

**Authors:** 


*Officers of N. Y. State Medical Society.*—At the last annual meeting of the State Medical Society, held at Albany on the 1st and 2d ult., Dr. Alex. H. Stevens, of New-York city, was elected President; Dr. Alex. H. Thompson, Vice President; Dr. P. Van Buren, Secretary; Dr. P. Van Olinda, Treasurer. Censors of the Southern District, Drs. E. G. Ludlow, J. C. Cheesman, Jas. R. Manley. Dr. C. R. Gilman is one of the Committe of Correspondence. The following were chosen delegates to the National Medical Association: Drs. Dyer Loomis, Augustus Willard, John McCall, P. H. Hard, S. Sprague, Robt. G. Frary, Brinsmade, Darius Clark, Naudain, Delafield, Gordon Buck, Beadle, Purple, Maltby Strong, Alex. Thomson, H. Burnell, Geo. W. Bradford, Enos Barnes. We learn that the meeting was well attended and spirited. We shall give a fuller account of the proceedings when they are officially published.

